# Social and geographic disparities in access to reference care site for patients with colorectal cancer in France

**DOI:** 10.1038/sj.bjc.6602571

**Published:** 2005-05-10

**Authors:** O Dejardin, A-M Bouvier, C Herbert, M Velten, A Buemi, P Delafosse, N Maarouf, S Boutreux, G Launoy

**Affiliations:** 1 ‘Cancers & Populations’ ERI3 INSERM Faculté de Médecine 14032 Caen Cedex, France; 2 FRANCIM (French network of cancer registries)

**Keywords:** geographic, disparities, colorectal cancer

## Abstract

The aim of this study was to investigate the relationship between social and geographic characteristics and the type of care centre for initial colorectal surgery in France. Patients living far from a reference cancer site were less frequently treated in a reference cancer site than those who were living near a reference cancer site OR_a_=(0.50 (0.33–0.76)). As for topography and emergency presentation, place of residence (urban/rural), occupation and marital status were not associated with the type of the care centre. Improvements in diagnosis and treatment and of clinical practice guidelines are therefore crucial to ensure equality in health care in France.

As suggested by the Calman–Hine report on cancer management (Calman and Hine, 1995), health authorities in all countries are faced with the difficulty of ensuring equal access to ‘high-quality, safe and effective treatment’ in a cancer care unit for all patients, irrespective of their social characteristics and place of residence.

For colorectal cancer, relative survival tends to be better in France than in other European countries for colon cancer (France 5 years colon cancer relative survival: 53%; European mean: 48%) and for rectum cancer (France 5 years rectum cancer relative survival: 49%; European mean: 44%) (Berrino *et al*, 1999). Several recent European studies suggest that such a goal has not yet been achieved since they have evidenced that low social class cancer patients in rural areas have a poorer survival rate than higher social class patients in urban areas (Auvinen and Karjalainen, 1997). France is the European country where social disparity in mortality is the greatest (Mackenbach *et al*, 1997). Several cancer-related studies have already evidenced that patient survival depends on social characteristics even after adjustments for age, cancer stage and tumour site. Colorectal cancer survival appeared poorer in farmers for both men and women, for women without occupation (Desoubeaux *et al*, 1997) and for patients living in houses with no comforts (Monnet *et al*, 1993).

The mechanisms underlying this social disparity tend to vary from one country to another and are not yet well known. Several studies suggest that differences in access to a specialised care centre are likely to contribute towards such a disparity. In Scotland, two studies (Pitchforth *et al*, 2002; Kingsmore *et al*, 2004) have evidenced that patients treated in a nonspecialist cancer unit were not given the same treatment as those treated in a specialist cancer unit, for colorectal cancer and breast cancer, respectively. Two other recent studies (Smith *et al*, 2003; McArdle and Hole, 2004) confirmed that colorectal cancer patients had a better survival rate when managed by specialised surgeons. Although these studies appear to establish the influence of the degree of specialisation on survival, the relationship between the social and geographic environment and the place of treatment remains unexplored in France. Regarding the Scottish results, it is important to determine whether there are any social or geographic ‘barriers’ against access to a specialist care unit.

To investigate the relationship between social or geographic factors and the degree of specialisation for initial surgery in France, we analysed data collected for all colorectal cancer patients diagnosed in 1995 in six French counties covered by a cancer registry (Calvados, Côte d'Or, Isère, Manche, Bas-Rhin and Haut-Rhin).

## MATERIALS AND METHODS

### Population

The six local cancer registries belong to the French network of cancer registries (FRANCIM). As such, their quality is regularly assessed (every 4 years) by French health authorities and the French Institute of Health and Medical Research (INSERM). Their quality is also regularly checked by the International Agency for Research on Cancer (IARC).

Between 1 January 1995 and 31 December 1995, the six French local registries collected 1535 cases of colorectal cancer. Owing to missing values, 122 patients were excluded (Table 1).

French public health authorities have defined a reference site as one or several care centres able to manage serious pathologies with bad prognosis and rare pathologies. Moreover, this care centre must propose specialised therapies making use of particular techniques. Actually, such care sites, mainly represented by University hospitals and regional comprehensive cancer centres, were exclusively pooled in regional capitals.

Using regional health care planning, the place of treatment was classified either as reference sites or as the other sites. Social and geographic variables included occupation, marital status, place of residence (rural/urban) and road-distance from the place of residence to the nearest reference cancer site. Occupation was pooled into four socio-professional categories (SPC) based on the classification of the French National Institute for Statistics and Economic Studies (INSEE level1 second edition, 1994): Class A: farmers; Class B: managers, executives and self-employed (including mainly craftspeople, shopkeepers and company directors); Class C: employees and workers (including farm labourers); Class D: without any occupation. Retirees were classified according to their longest former occupation. Occupation was unknown for one-third of the patients (*N*=460). The place of residence was classified as urban or rural according to the official classification of INSEE (‘Study zoning’). Road-distance between the place of residence and the nearest reference cancer sites was calculated using the CHRONOMAP 2.1 software (Magellan ingénierie) combined with MAPINFO 6.5 (MapInfo Corporation) (mean value=46.4 km). The other variables were: cancer stage (Dukes A, B, C, metastasis or not operable), topography (colon *vs* rectum), age, gender and emergency presentation (Yes/No) according to main symptom at diagnosis (intestinal obstruction, perforation, peritonitis).

Two counties (Manche and Haut-Rhin) in our study were not equipped with a reference cancer site. Nevertheless, patients of these two counties could have access in a reference cancer care in neighbouring counties. In Haut-Rhin, two general hospitals have a high degree of specialisation in cancer treatment, as in reference cancer level. In order to take into account this particular situation, we conducted complementary analysis without this county.

### Statistical analysis

The probability of being referred to a reference cancer site for initial surgical management (curative or palliative surgery) was analysed using a logistic regression model with the SAS software (SAS Institute Inc., Cary, NC, USA). Age, gender and cancer stage were systematically included in multivariate analysis. They were considered as adjustment variables.

## RESULTS

Only a few patients (22.6%) were treated for initial surgery in a reference cancer site (Table 2). The type of site strongly depended on the road-distance to the nearest reference cancer site. Patients living far from a reference cancer site (more than 46.4 km) were less frequently treated in a reference cancer site than those who were living near a reference cancer site (less than 46.4 km) (adjusted OR, OR_a_=(0.50 (0.33–0.76)). Only four patients in the Haut-Rhin, a county without any reference cancer site, were operated in a reference cancer site with the result that the corresponding OR was not calculated in the table. The influence of road-distance on access to reference site remained significant even when data from Haut-Rhin were excluded (OR_a_=(0.50 (0.33–0.76)). When the counties without a reference site (Manche and Haut-Rhin) were grouped together, the OR_a_ was 0.50 (0.30–0.82), compared to those equipped with. Female patients were less often treated in a reference cancer site than male patients (OR_a_=(0.75 (0.57–0.98)). As for topography and emergency presentation, place of residence (urban/rural), occupation and marital status were not associated with the type of the care centre. Age and cancer stage at diagnosis were not associated with the kind of care centre but were forced into the model.

Moreover, road-distance was not significantly associated with the type of care centre for males (OR_a_=0.61 (0.36–1.04)), and strongly associated for females (OR_a_=0.37 (0.19–0.73)). Road-distance was significantly associated with type of care centre for elderly (more than 75 years) patients (OR_a_=0.30 (0.14–0.67)), but not for youngest (less than 75 years) (OR_a_=0.63 (0.39–1.03)).

## DISCUSSION

Our study suggests that access to a reference cancer site in France is strongly determined by the distance between the place of residence and the nearest reference site. On the other hand, social characteristics such as occupation, marital status or place of residence (urban/rural) have no influence on the kind of health care centre for initial surgery, suggesting that the difference of management for people living far from a reference cancer site is more due to the geographical distance than to a social distance.

Moreover, our results seem to demonstrate that the influence of geographical surrounding is different according to gender and age. A previous French population-based study already demonstrated that patients living in rural areas had a worse survival than those living in urban areas, especially for women (Launoy *et al*, 1992).

The lack of information on occupation represents the main limitation of our study in which the occupation was unknown for one-third of the population, more often for female patients (39.8%) than for male patients (21.5%), and less often for patients treated in reference centres (23.9%) than in the others (35.0%). Such a lack of information is unavoidable in French cancer registries since the relevant data do not appear systematically in the medical file. This reduces considerably the robustness of the study and might introduce a bias.

Since a relationship between type of centre for initial surgery and survival is not yet established in France, the influence of the distance between the place of residence and the nearest reference health care centre on the access to a reference cancer site does not mean that patients living far from a reference health care centre do not receive ‘high-quality, safe and effective treatment’. Taking into account the high mean age of colorectal cancer patients (69.9 years), it merely reflects, particularly for women and for elderly patients, a preference for proximity due to a reduction in the mobility of patients.

However, if further French studies conducted on large populations establish a difference in survival according to type of cancer care health centre, the geographical disparities in access to reference cancer site established in our study would imply geographical inequalities in cancer care management as in a recent English study (Kim *et al*, 2000), especially for women and elderly patients.

In any case, since the geographical distance is a major obstacle in the access to reference care centre in France, the preservation of high quality of care in nonreference centres by dissemination of improvements in diagnosis and treatment and of clinical practice guidelines is therefore crucial to ensure equality in health care.

## Figures and Tables

**Table 1:** Colorectal cancer patients diagnosed in 1995 in six French counties

	**Reference cancer site**		**Others**		**Total**	
	***N*=306**		***N*=1107**		***N*=1413**	
	*N*	%	*N*	%	*N*	%
*Age*						
<65 years	94	30.72	330	29.81	424	30.01
65–74 years	113	36.93	387	34.96	500	35.39
75–84 years	76	24.84	274	24.75	350	24.77
>84 years	23	7.52	114	10.30	137	9.70
Unknown	0	0.00	2	0.18	2	0.14

*Gender*						
Male	187	61.11	577	52.12	764	54.06
Female	119	38.88	530	47.87	649	45.93

*Topography*						
Colon	184	60.13	681	61.52	865	61.21
Rectum	122	39.97	426	38.48	548	38.78

*Type of sites*						
Urban	190	62.09	682	61.61	872	61.71
Rural	116	37.91	424	38.30	540	38.22
Unknown	0	0.00	1	0.09	1	0.07

*Marital status*						
Married	82	26.80	328	29.63	410	29.02
Singled	183	59.80	554	50.05	737	52.16
Unknown	41	13.40	225	20.33	266	18.83

*Social class* *						
Class A	31	10.13	104	9.39	135	9.55
Class B	68	22.22	203	18.34	271	19.18
Class C	103	33.66	306	27.64	409	28.95
Class D	31	10.13	107	9.67	138	9.77
Unknown	73	23.86	387	34.96	460	32.55

*Cancer stage*						
Dukes A, B or C	229	74.84	874	78.95	1103	78.06
Metastasis and not operable	67	21.90	191	17.25	258	18.26
Unknown	10	3.27	42	3.79	52	3.68

*County*						
Calvados	80	26.14	255	23.04	335	23.71
Côte-d'Or	35	11.44	117	10.57	152	10.76
Isère	43	14.05	167	15.09	210	14.86
Manche	35	11.44	203	18.34	238	16.84
Bas-Rhin	109	35.62	186	16.80	295	20.88
Haut-Rhin	4	1.31	179	16.17	183	12.95

*Road-distance to nearest reference cancer site (km)*						
<46.4	235	76.80	563	50.86	798	56.48
>46.4	71	23.20	539	48.69	610	43.17
Unknown	0	0.00	5	0.45	5	0.35

*Type of presentation*						
Screening, Symptom	258	84.31	906	81.84	1164	82.37
Emergency	44	14.38	148	13.37	192	13.59
Unknown	4	1.31	53	4.79	57	4.03

* Class A: farmers; Class B: managers, self-employed; Class C: employees and workers; Class D: without any occupation.

**Table 2:** Determinants of management in reference cancer site (logistic regression-final model)

	**Other**	**Reference care site**				
	***N*=1107**	***N*=306**				
	***N***	***N***	**Adjusted odds ratio**	**95% confidence interval limits**		***P*-values**
Age						
<65 years	330	94	1.00			*P*=0.855
65–74 years	387	113	1.05	0.76	1.46	
75–84 years	274	76	0.96	0.67	1.37	
>84 years	114	23	0.78	0.46	1.33	
Unknown	2	0	Not calculated			

*Gender*						*P*=0.034
Male	577	187	1.00			
Female	530	119	0.75	0.57	0.98	

*Cancer stage*						*P*=0.198
Dukes A, B or C	874	229	1.00			
Metastasis or not operable	191	67	1.30	0.93	1.81	
Unknown	42	10	0.75	0.36	1.54	

*County*						*P*<0.001
Calvados	255	80	1.00			
Côte d'or	117	35	0.94	0.59	1.49	
Isère	167	43	0.81	0.53	1.25	
Manche	203	35	0.93	0.53	1.62	
Bas-Rhin	186	109	1.74	1.22	2.47	
Haut-Rhin	179	4	Not calculated			

*Road-distance to nearest reference care site (km)*						*P*=0.005
<46.4	563	235	1.00			
>46.4	539	71	0.50	0.33	0.76	
Unknown	5	0	Not calculated			
